# Calcium-Alkali Syndrome in the Modern Era

**DOI:** 10.3390/nu5124880

**Published:** 2013-11-27

**Authors:** Ami M. Patel, Gbemisola A. Adeseun, Stanley Goldfarb

**Affiliations:** 1 Division of Nephrology and Hypertension, College of Medicine, Drexel University, Philadelphia, PA 19102, USA; 2 Division of Nephrology, Keck School of Medicine, University of Southern California, Los Angeles, CA 90033, USA; E-Mail: adeseun@usc.edu; 3 Division of Renal-Electrolyte and Hypertension, Perelman School of Medicine, University of Pennsylvania, Philadelphia, PA 19104, USA; E-Mail: stanley.goldfarb@uphs.upenn.edu

**Keywords:** calcium-alkali syndrome, milk-alkali syndrome, hypercalcemia

## Abstract

The ingestion of calcium, along with alkali, results in a well-described triad of hypercalcemia, metabolic alkalosis, and renal insufficiency. Over time, the epidemiology and root cause of the syndrome have shifted, such that the disorder, originally called the milk-alkali syndrome, is now better described as the calcium-alkali syndrome. The calcium-alkali syndrome is an important cause of morbidity that may be on the rise, an unintended consequence of shifts in calcium and vitamin D intake in segments of the population. We review the pathophysiology of the calcium-alkali syndrome.

## 1. History of the Milk-Alkali Syndrome

In 1915, Bertram Welton Sippy introduced a cocktail to treat peptic ulcer disease with hourly administration of milk and cream with Sippy powders, which were a mixture of sodium bicarbonate with calcinated magnesia or bismuth subcarbonate [1]. With the Sippy diet, about 32 g of sodium bicarbonate and 10–32 g of calcium carbonate (4 to 13 g of elemental calcium) were administered daily [2]. As Sippy’s treatment became prevalent, some patients who were treated with high doses of calcium and sodium bicarbonate developed a constellation of symptoms including headache, nausea, vomiting, light-headedness, anorexia, distaste for milk, musculoskeletal pains, weakness, dizziness, mental clouding, and renal failure [2,3]. Later, in 1949, Burnett *et al.* [4] described these symptoms as part of “milk and alkali syndrome”. The most common findings were hypercalcemia, metabolic alkalosis, renal insufficiency, and soft tissue calcium deposits.

Three different progressive stages have been described. First, there is an acute form, referred to as “toxemia”, which occurs 2–30 days after calcium ingestion. This stage has been characterized by irritability, vertigo, apathy, headaches, weakness, myalgias, and vomiting. Second, the intermediate phase, called “Cope’s syndrome,” includes symptoms of the acute form plus conjunctivitis. Last is the chronic form, or Burnett’s syndrome, which is characterized by soft tissue calcification including conjunctivitis, band keratopathy of the cornea, musculoskeletal deposits, and nephrocalcinosis [5].

## 2. Emergence of the Calcium-Alkali Syndrome

In the 1970s, the incidence of milk-alkali syndrome fell dramatically as the use of antacids decreased with the introduction of histamine blockers. The 1990s were marked by a resurgence of the milk-alkali syndrome, in large part due to the widespread use of calcium and vitamin D supplementation among post-menopausal women for the treatment and prevention of osteoporosis, which affects more than 10 million individuals in the United States [6,7]. Current recommendations for treatment of osteoporosis are 1200 mg of calcium and 800–1000 IU of vitamin D [7,8].

The modern version of milk-alkali syndrome is now known as calcium-alkali syndrome. This evolution in terminology reflects the current pathogenesis of the disorder, which is related to excess calcium supplementation or calcium containing antacids [9]. Calcium-alkali syndrome is now considered one of the leading causes of hospital admission for hypercalcemia [10]. The true incidence of calcium alkali syndrome is unknown. In a recent retrospective study at a single center, from 1998 to 2003, calcium-alkali syndrome was the third most common cause of hypercalcemia (8.8%) and second most common cause of severe hypercalcemia (>14 mg/dL). About 34% of the patients had malignancy and 30% had primary hyperparathyroidism [11]. Many of the patients reported consuming less than 2 g of elemental calcium per day in the form of calcium carbonate. With the caveat that self-reported calcium ingestion may not be accurate, the amount described is much lower than the usual minimum 4 g of calcium intake that was previously associated with the milk-alkali syndrome [11]. The lower threshold for calcium intake associated with calcium-alkali syndrome may be due to increased vitamin D intake resulting in enhanced intestinal calcium absorption.

## 3. Populations at Risk

Whereas the traditional milk-alkali syndrome affected younger male patients with peptic ulcer disease [12,13], the demographics have changed to post-menopausal women, solid organ transplant recipients, pregnant women, bulimic patients, and those on dialysis [6,11,12,13,14]. Post-menopausal women and solid organ transplant recipients are encouraged to take calcium supplementation along with vitamin D for the prevention and treatment of osteoporosis. Post-menopausal women also have decreased estrogen which results in bone loss and decreased calcium absorption. Estrogen may also upregulate the transient receptor potential vanilloid member 5 (TRPV5), a channel that is critical in calcium uptake in the kidney, resulting in enhanced calcium-reabsorption [15]. Thus, lack of estrogen in and following the menopausal transition may result in a rise in obligatory urinary calcium loss, reduced intestinal calcium absorption, negative calcium balance, and increased risk of osteoporosis [16].

Elderly patients are at risk of developing calcium-alkali syndrome relating to aging bone metabolism and reduced renal function, leading to impaired bone mineralization and reduced calcium excretion, respectively. Pregnant women are susceptible to calcium-alkali syndrome due to hyperemesis, which causes volume depletion and metabolic alkalosis, and increased calcium absorption through the gastrointestinal tract, which is thought to be mediated by prolactin, placental lactogen, and increased 1,25-dihydroxyvitamin D [17]. Bulimic patients are a high-risk group for developing calcium-alkali syndrome related to metabolic alkalosis that is associated with vomiting and diuretic abuse, as well as poor eating habits. In the Far East, Asia, and South Pacific, calcium-alkali syndrome has been reported in betel nut chewers. Worldwide, betel nut is used by an estimated 600 million people [18]. The lime paste added to the nut is made from ground oyster shells which consist of calcium oxide and calcium hydroxide [19].

## 4. Physiology

The integral features of calcium-alkali syndrome are excess ingestion of calcium and often absorbable alkali leading to the classic triad of hypercalcemia, metabolic alkalosis, and varying degrees of renal insufficiency [10,13,20]. Initially, the traditional milk-alkali syndrome was characterized by elevated serum phosphate concentration, probably secondary to the ingestion of phosphate-rich milk and cream in the Sippy diet [21]. In contrast, calcium-alkali syndrome is associated with hypophosphatemia or low-normal serum phosphorus level as a result of the phosphate-binding capacity of calcium carbonate and other calcium containing supplements resulting in decreased phosphate absorption. These effects are more pronounced in elderly patients and patients with eating disorders who have reduced dietary phosphate and protein intake [6]. The vast majority of cases are associated with calcium carbonate supplements [10].

**Figure 1:**
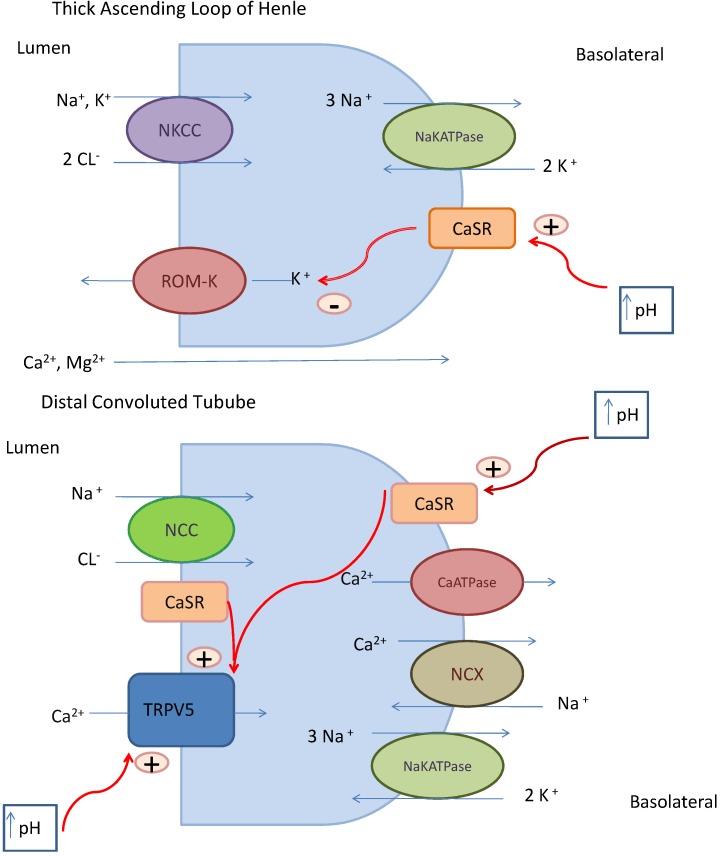
Mechanisms of renal calcium transport [9]. In the thick ascending limb of Henle’s loop, the creation of a lumen positive voltage tends to drive Ca^2+^ and Mg^2+^ through a paracellular path and thereby produces a high rate of reabsorption of the divalent ions. The CaSR modulates this transport activity by altering rates of K efflux and thereby contributing to the lumen positive voltage. In the distal convoluted tubule, a three-step process facilitates active and transcellular Ca^2+^ transport. The first step is entry of luminal Ca^2+^ at the apical side of the cell through the TRPV5 channel. Subsequently, calbindin, a calcium binding protein buffers Ca^2+^ and the Ca^2+^ diffuses to the basolateral membrane. At the basolateral membrane, Ca^2+^ is extruded by across the basolateral membrane by NCX. This process is controlled by calciotropic hormones, including parathyroid hormone and 1,25(OH)2D3. Abbreviations: NKCC, sodium potassium-2-chloride co-transporter; ROM-K, renal outer medullary potassium channel; CaSR, calcium sensing receptor; NaKATPase, sodium potassium ATPase; +, stimulates; −, inhibits; NCC, sodium chloride co-transporter; NCX, sodium calcium exchanger; TRPV5, transient receptor potential vanilloid membrane calcium channel. (Note: Permission obtained from [9], Copyright American Society of Nephrology.)

The pathogenesis of calcium-alkali syndrome is intricate and involves the interplay of multiple systems including intestine, kidney, and bone [6,22]. An average healthy adult contains about 1 kg of calcium (or 25,000 mmol) with over 99% located in the bone and less than 1% (20 mmol) in the extracellular fluid [22]. The kidney plays an important role in calcium balance. Fine-tuning of calcium reabsorption occurs in the distal convoluted tubule of the nephron under the influence of several factors, including parathyroid hormone, calcium, calcitriol, and calcitonin. Nevertheless, the rate-limiting step in renal calcium reabsorption is the TRPV5 calcium channel activity and to a lesser extent, TRPV6, both located on the luminal side of the distal tubule. Through TRPV5, calcium enters the cell, is carried across the cell cytoplasm by calbindin D23. Calcium ions then exits through a sodium-calcium exchanger or calcium ATPase pump located on the basolateral side (Figure 1) [6].

Hypercalcemia occurs when the influx of calcium into extracellular fluid exceeds the excretion by the kidney [22]. Calcium-alkali syndrome can be divided into generation and maintenance phases. In the generation phase, the intestinal component is the most important since it is the site of calcium absorption. Factors affecting the amount of calcium absorption include dietary intake, vitamin D level, and acidity. An acidic environment increases the availability of free calcium for absorption in the gut, predominantly in the proximal small intestine through a combination of active vitamin D-dependent transport (saturable) and passive unregulated paracellular diffusion [6,16]. Therefore, meals that raise gastric acidity such as animal protein and carbohydrate-based meals are associated with increased calcium absorption [6]. The threshold amount of calcium generally considered to predispose to calcium-alkali is above 4 g; however, there are reports of calcium-alkali with as little as 1–1.5 g of calcium supplementation, a dose that is consistent with many dietary guidelines [11,13,23].

Once calcium is absorbed into the extracellular fluid, uptake occurs in the bone envelope but more slowly in structural bone [24]. However, following post-epiphyseal closure, the ability of bone to buffer calcium loads diminishes. In the young adult and child, calcium preferentially deposits in the bone with high intake and renal calcium excretion is minimal to allow a positive calcium balance for growth. However, in the aging individual, the net flux of calcium is out of bone, making bone less available for buffering excessive amounts of calcium [22]. Thus, the elderly are more susceptible to the calcium-alkali syndrome. Natural protective mechanisms against the development of calcium-alkali syndrome also operate in elderly individuals, including decreased intestinal absorption of calcium, decreased sensitivity to vitamin D supplementation, and reduced gastric acidity [6].

In the event that hypercalcemia occurs, counter-regulatory mechanisms re-establish normocalcemia. First, the high calcium level suppresses parathyroid hormone (PTH) secretion, which reduces bone efflux of calcium. A decreased PTH concentration also functions to suppress calcitriol production, resulting in decreased intestinal absorption of calcium. Despite these modifying mechanisms, other features of the calcium-alkali syndrome may further perpetuate the syndrome. Hypercalcemia-mediated renal vasoconstriction causes a decline in glomerular filtration rate, filtered load of calcium, and ultimately, reduced calcium excretion in a self-propagating cycle [13]. Furthermore, an enhanced reabsorption of any filtered calcium in the proximal tubule operates via a paracellular pathway through solvent drag in addition to enhanced reabsorption of bicarbonate. Alkaline tubular pH also sensitizes the TRPV5 channel, resulting in enhanced calcium absorption in the distal tubule (see Figure 1) [6].

Hypercalcemia affects water and salt conservation. Hypercalcemia causes a furosemide-like effect by inhibiting secretory potassium channel activity via actions initiated by the calcium-sensing receptor (CaSR) in the thick ascending limb of Henle’s loop (Figure 1). Without the luminal positive voltage caused by potassium efflux, the voltage driving force is obliterated and there is less sodium chloride reabsorption through the sodium potassium-2-chloride co-transporter (NKCC) channel [18]. Metabolic alkalosis enhances the sensitivity of the CaSR to hypercalcemia. Moreover, hypercalcemia causes nephrogenic diabetes insipidus by inhibiting vasopressin action at the V2 receptor in the collecting duct [25]. These mechanisms lead to salt and water losses and, therefore, they contribute to volume depletion which further stimulates proximal tubular calcium reabsorption. Over time, chronic renal ischemia induced by the calcium-alkali syndrome can result in irreversible renal dysfunction. In addition, renal dysfunction can occur as a result of calcium precipitation in the kidney, manifested as nephrocalcinosis. Calcium deposits may also develop in other tissues, such as the vasculature, cardiac valves, and the cornea [13].

Commonly used medications can influence calcium retention, predisposing some individuals to the development of calcium alkali syndrome. Thiazide diuretics directly enhance calcium absorption in the proximal tubule of the nephron by promoting volume depletion and subsequent metabolic alkalosis. Metabolic alkalosis favors calcium absorption, as described previously. Agents that decrease glomerular filtration rate can predispose to calcium-alkali by decreasing calcium excretion. Inhibitors of the renin-angiotensin system, widely used for the treatment of hypertension, cardiovascular disease, and renal disease, can result in a decline in glomerular filtration rate. Non-steroidal anti-inflammatory drugs such as ibuprofen and naproxen, can also decrease glomerular filtration rate and subsequent calcium excretion via their inhibitory effects on prostaglandins [6]. Consumption of absorbable alkali such as aluminum hydroxide and magnesium hydroxide, components of over-the-counter dyspepsia remedies, can also increase tubular calcium reabsorption in the kidney via their effects on pH. With an increase in pH, the affinity of the calcium-sensing receptor for calcium is increased which initiates a cascade of events that promotes calcium reabsorption in both the proximal and distal tubule of the nephron (see Figure 1) [9].

## 5. Role of Parathyroid Hormone and 1,25-Hydroxyvitamin D

Calcium-alkali syndrome is often confused with hyperparathyroidism as both may have combined hypercalcemia and hypophosphatemia. The PTH concentration may help distinguish between the two, but assay differences may obscure a correct diagnosis. The amino-terminal assays are considered sensitive and specific, but some studies have shown rather poor discrimination due to the short half-life of the fragment and episodic secretion of the PTH. While the carboxyl-terminal assay was initially thought to be superior, any decrease in renal function causes accumulation of an increased concentration of carboxyl fragments. Approximately 10% of reported cases of patients with milk-alkali syndrome underwent unnecessary parathyroid exploration [10,26].

While serum concentrations of 1,25-hydoxyvitamin D are elevated in primary hyperparathyroidism, the levels of 1,25-hydroxyvitamin D are generally low in calcium-alkali syndrome because PTH is suppressed. However, reports exist of 1,25-hydroxyvitamin D levels that are not suppressed, perhaps identifying a subset of patients that are at high risk of developing calcium-alkali syndrome [10].

## 6. Role of Supplemental Calcium Intake

On average, men and women above the age of 50 years consume about 750–950 mg of elemental calcium per day in their diet. About 60% of the ingested calcium comes from dairy products [27]. Around 40% of the U.S. population, and almost 70% of older women, use calcium-containing dietary supplements [27]. Due to the widespread use of calcium supplementation for osteoporosis prevention and treatment, about 5% of women older than 50 years have an estimated calcium intake that exceeds the Tolerable Upper Intake Level for calcium (established by the Institute of Medicine, Washington, DC, USA [28]) by a substantial amount each day, *i.e.*, total calcium intake may exceed 2300 mg per day, according to NHANES (National Health and Nutrition Examination Survey, Centers for Disease Control and Prevention, Atlanta, GA, USA) data from 2003 to 2006 [27,29]. Chronic exposure to high calcium intake can lead to a positive calcium balance, especially in the elderly, because of reduced renal function with resultant hypocalciuria and aging bone metabolism with a decreased capacity to take up the calcium load.

The beneficial effect of calcium supplements on the prevention of osteoporotic fracture is modest. In a meta-analysis by Bischoff-Ferrari *et al.* [30], hip fracture risk was not reduced significantly with increased calcium intake from 800 to 1600 mg (RR 0.92 with 95% CI 0.81–1.05). Furthermore, calcium carbonate and citrate can reduce phosphate absorption, which may be detrimental for bone mineralization. Therefore, the benefits of calcium supplementation on osteoporosis should be weighed against the potential risks. In most cases, calcium intake is not rate-limiting for bone formation and modest improvements in bone mineral density is not accompanied by reduction in rates of bone fracture. According to the Institute of Medicine, risk of harm increases when calcium intake surpasses 2000 mg per day and the vitamin D intake is greater than 4000 IUs per day [28]. As per the U.S. Preventive Services Task Force (Rockville, MD, USA), evidence is insufficient for the benefit beyond 400 IU of vitamin D3 and more than 1000 mg of calcium for the primary prevention of fractures in non-institutionalized postmenopausal women [31]. According to the National Osteoporosis Foundation (Washington, DC, USA), dietary calcium intake in excess of 1200–1500 mg may increase the risk for cardiovascular disease or kidney stones [8]. Table 1 shows the Recommended Dietary Allowances for calcium and vitamin D intake by age groups [28].

**Table 1:** Recommended dietary allowances (RDAs) for calcium and vitamin D [28].

Age	Calcium (mg)	Vitamin D (IU)
*Infants*		
0–6 months	200	400
6–12 months	260	400
1–3 years	700	600
4–8 years	1000	600
9–18 years	1300	600
19–50 years	1000	600
Pregnancy	1000	600
*51–70 Years*		
Male	1000	600
Female	1200	600
Greater than 70 years	1200	800

The quantity of elemental calcium contained in various popular calcium supplements is summarized in Table 2 [9]. Calcium carbonate is more commonly available and is inexpensive. It contains about 40% of elemental calcium and requires stomach acid for absorption; therefore, calcium carbonate should be taken with meals. On the other hand, calcium citrate is considered the better absorbed calcium supplement because it does not require extra acid for absorption and can be taken on empty or full stomach [32]. However, it contains 21% elemental calcium [29].

**Table 2:** Amount of elemental calcium in various supplements [9]. Caltrate^®^ (Pfizer, Kings Mountain, USA); Centrum^®^ (Pfizer, Kings Mountain, NC, USA); Rolaids^®^ (Chattem Inc., Chattanooga, TN, USA); Os-Cal^®^ (GlaxoSmithKline, Brentford, UK); Tums^®^ (GlaxoSmithKline, Brentford, UK); Tums Ultra^®^ (GlaxoSmithKline, Brentford, UK); Viactiv^®^ (McNeil Nutritionals, Fort Washington, DC, USA); Citracal^®^ (Bayer, Pittsburg, PN, USA); Phoslo^®^ (Fresenius Medical Care North America, Waltham, MA, USA). (Note: Permission obtained from [9], Copyright American Society of Nephrology).

Type	Trade Name	Elemental Calcium (mg)	Vitamin D (IU)
Calcium carbonate	Caltrate^®^	600	400 400 800
	Centrum^®^	200	
	Centrum^®^ Ultra Women’s	500	
	Rolaids^®^ Extra Strength	471	
	Os-Cal^®^	500	
	Tums^®^	200	
	Tums Ultra^®^	400	
	Viactiv^®^	500	
Calcium citrate	Citracal^®^ Regular with Vit D	250	200
Calcium acetate	Phoslo^®^	167	-

Calcium supplements can raise serum calcium levels to a modest degree. Changes in serum calcium level are often reflected by opposite but magnified and sustained responses in the PTH level [32]. An acute load of calcium above 400 mg has been shown to suppress serum PTH secretion for as long as 8–10 h [33]. PTH changes sometimes occur before a calcemic response is detected [32]. On a 1300 mg calcium diet for one week, fasting serum calcium level increased by 3% in one small study by Martini *et al.* [34]. 1 g of calcium as either citrate or as lactate-gluconate raised the mean ionized calcium level from 1.22 to 1.30 mmol/L [35]. The less absorbable calcium carbonate increased ionized calcium by approximately 0.05 mmol/L, and the same calcium load as bone meal only raised it by 0.03 mmol/L. In comparison, dairy products increased ionized calcium by only one-sixth of the level seen following consumption of soluble calcium supplement with comparable calcium content [35]. Ingesting 1200 mg calcium as fortified skim milk increased the total (not ionized) serum calcium level by only 0.03 mmol/L [35]. However, in another study by Martini *et al.* [34] involving 12 subjects, no difference was observed in the postprandial total calcium level and the degree of PTH reduction between orange juice fortified with calcium-citrate malate, skim milk, or calcium carbonate. Equal doses of calcium carbonate and calcium citrate produced a similar increment in total serum calcium and reduction in the PTH level [36]. In a study by Hanzlik *et al.* [32], 1200 mg calcium carbonate produced a serum calcium rise of about 4% and decreased the PTH level by about 20%–40%; nevertheless, these changes did not differ significantly from placebo. Administration of similar dose of calcium citrate caused the serum calcium to rise about 9% (*p* = 0.001 [32]. In summary, varying effects on the serum calcium and PTH concentrations result from calcium supplementation compared to dietary calcium intake from food, and certain calcium supplements, such as calcium formate, have even a more pronounced elevation of serum calcium than other supplements [36].

In general, ingestion of equivalent doses of calcium from dairy products has a smaller effect on serum calcium level than calcium supplements, which may explain the lack of detrimental vascular effect by dietary calcium intake as found in observational studies [35,37,38]. Calcium in food is less bioavailable because of the presence of calcium-binding agents, such as oxalic acid, phytates, fiber, and phosphate. Oxalic acid, which exists in some plant foods, binds to calcium directly, making calcium less available for absorption. Foods with high amounts of oxalic acid such as spinach, collard greens, sweet potatoes, rhubarb, and beans actually permit little calcium absorption. Phytates can also bind to the calcium, decreasing its absorption. Foods high in phytates include whole-grain products, wheat bran, beans, seeds, nuts, and soy isolates. Unlike oxalic acid, phytates will bind the calcium from other food sources consumed at the same meal. Furthermore, fibers such as in wheat bran, can also bind to the calcium in the intestine and decrease its absorption [16,29]. Individuals may also ingest medications with phosphate binding properties such as aluminum hydroxide, increasing the bioavailability of calcium for absorption [39].

Due to stimulation of active enteric absorption of calcium by 1,25-dihydroxyvitamin D, a higher percentage of calcium is absorbed from smaller doses of calcium (below 500 mg) than from larger calcium loads. Increases in serum ionized calcium were similar after loads of 1000 and 2000 mg calcium, which results from saturation of the vitamin-D mediated active mechanism of calcium absorption in the intestinal tract [33]. As calcium intake increases, net absorbed calcium increases steeply until active transport becomes saturated, after which the percentage of calcium absorbed drops. Therefore, the peak absorption of calcium occurs at around a 400 mg calcium load and then it levels off. Absorption of calcium may be as high as 60% at very low intake and as low as 20% at a high intake [40]. 1,25-Dihydroxyvitamin D (hormonal form) may also mediate enteric calcium absorption [41], and according to the NHANES 2003–2006 data, 37% of the U.S. population used a vitamin D supplement [27].

## 7. Long-Term Sequelae of Calcium Supplementation

Chronic ingestion of moderate to high doses of calcium supplementation has been found to have variable effects on cardiovascular morbidity and mortality. In a study by Wang *et al.* [42], 1471 healthy post-menopausal women were randomized to calcium 600 mg or 1200 mg per day. Although increased serum calcium and phosphate levels were associated with aortic calcification in elderly women, neither dietary calcium intake nor calcium supplementation was associated with changes in aortic calcification in this trial [42]. In the Women’s Health Initiative study involving nearly 36,000 postmenopausal women followed for seven years randomized to 1 g of calcium carbonate supplementation with 400 IU vitamin D, there was no increase in cardiovascular events (HR 1.04, 95% CI 0.92–1.18) [43]. On the other hand, a meta-analysis of 15 trials by Bolland *et al.* [38] found that calcium supplementation of 500 mg or greater was associated with hazard ratio of 1.31 for myocardial infarction (95% CI 1.02–1.67, *p* = 0.035). The authors of this meta-analysis concluded that the number needed to treat with calcium for five years to cause one event was 69 for myocardial infraction, 100 for stroke, and 77 for death while the number needed to treat to prevent one fracture was 39 [38]. Interestingly, supplemental calcium treatment was associated with increased risk of myocardial infarction in people with dietary intake above the median of 805 mg/day (HR 1.85, 95% CI 1.28–2.67), but there was no increased risk in those with dietary intake intakes below the median (HR 0.98, 95% CI 0.69–1.38). This finding suggests that a threshold may exist above which combined dietary and supplemental calcium may result in increased cardiovascular risk and that the higher risk may be due to higher calcium from supplements rather than from dietary calcium. This meta-analysis did not examine studies with vitamin D supplementation [38]. In a recent prospective study by Li *et al.* [44] of the Heidelberg cohort of 23,980 participants, dietary calcium did not raise the risk for cardiovascular disease; however, calcium supplementation was significantly associated with increased risk for myocardial infarction (HR 1.86, 95% CI 1.12–5.12). Again, this article indicates that calcium supplements rather than dietary calcium might confer increased cardiovascular risk.

Calcium supplements may contribute to vascular calcification by causing a positive calcium balance as evidenced by a reduction in PTH and a modest rise in serum calcium level. Serum calcium is positively correlated with carotid artery plaque thickness, aortic calcification, incidence of myocardial infarction, and mortality [45,46]. It is possible that an increase in serum calcium level from supplements induces vascular calcification by altering inhibitors such as fetuin-A, matrix Gla protein, pyrophosphate, osteoprotegerin, and bone morphogenic protein-7. It is important to recognize that 29 million Americans have stage 3 chronic kidney disease. In these individuals fetuin-A and matrix Gla protein are frequently reduced, thus, promoting the development and progression of vascular calcification [47]. In patients with renal failure, calcium supplements have been well established to worsen vascular calcification and increase mortality in both dialysis and pre-dialysis populations. Serum calcium can also directly bind to the calcium-sensing receptor that is expressed on vascular smooth muscle cells [35]. In a study by Alam *et al.* [39], exposing vascular smooth muscles cells *in vitro* to high calcium level caused increased mineralization.

## 8. Preventative Measures and Treatment

To avoid the calcium-alkali syndrome, it is generally recommended that total diet plus supplements) calcium intake be reduced to less than 1.2–1.5 g daily and to avoid absorbable alkali [6]. It is advisable to monitor calcium levels periodically in patients who are also receiving calcium and/or vitamin D supplementation. Once calcium-alkali syndrome occurs, the cornerstone of treatment is to reduce calcium intake and increase calcium excretion. Generally, calcium and vitamin D supplements are held in the short term. As many individuals with calcium-alkali are volume depleted, adequate volume resuscitation can serve to increase glomerular filtration rate and reverse metabolic alkalosis, both of which favor calcium excretion. Bisphosphonates are useful agents in extreme cases to decrease osteoclast activity in bone and to help lower the serum calcium level. Severe symptomatic hypercalcemia can be treated with dialysis using low calcium dialysate or continuous renal replacement therapy(CRRT) without calcium in the replacement fluids. Hypercalcemia usually resolvesover several days, although some evidence suggests that it can take up to six months [13]. Approximately one-third of the patients with calcium-alkali syndrome develop permanent impairment of renal function [10].

## 9. Conclusions

Calcium-alkali syndrome, an important clinical problem, has recently become more prevalent because shifts in the epidemiology of the disorder, *i.e.*, from previous patients with peptic ulcer disease to a modern cohort of calcium supplement users. In patients with osteoporosis, the view that fracture risk and calcium intake are inversely related has mistakenly led many osteoporotic patients to take excessive quantities of supplemental calcium. In particular, the concomitant supplemental intake of increased calcium and vitamin D predisposes high-risk groups to calcium-alkali syndrome. Judicious use of calcium and vitamin D supplements, along with careful monitoring, can reduce the long-term sequelae of calcium-alkali syndrome, including vascular calcification, adverse cardiovascular events, and renal insufficiency.
